# Autonomously Folding Protein Fragments Reveal Differences in the Energy Landscapes of Homologous RNases H

**DOI:** 10.1371/journal.pone.0119640

**Published:** 2015-03-24

**Authors:** Laura E. Rosen, Susan Marqusee

**Affiliations:** 1 Biophysics Graduate Group, University of California, Berkeley, CA, United States of America; 2 California Institute for Quantitative Biosciences – Berkeley, Berkeley, CA, United States of America; 3 Department of Molecular and Cell Biology, University of California, Berkeley, CA, United States of America; University of South Florida College of Medicine, UNITED STATES

## Abstract

An important approach to understanding how a protein sequence encodes its energy landscape is to compare proteins with different sequences that fold to the same general native structure. In this work, we compare *E*. *coli* and *T*. *thermophilus* homologs of the protein RNase H. Using protein fragments, we create equilibrium mimics of two different potential partially-folded intermediates (I_core _and I_core+1_) hypothesized to be present on the energy landscapes of these two proteins. We observe that both *T*. *thermophilus* RNase H (ttRNH) fragments are folded and have distinct stabilities, indicating that both regions are capable of autonomous folding and that both intermediates are present as local minima on the ttRNH energy landscape. In contrast, the two *E*. *coli* RNase H (ecRNH) fragments have very similar stabilities, suggesting that the presence of additional residues in the I_core+1_ fragment does not affect the folding or structure as compared to I_core_. NMR experiments provide additional evidence that only the I_core_ intermediate is populated by ecRNH. This is one of the biggest differences that has been observed between the energy landscapes of these two proteins. Additionally, we used a FRET experiment in the background of full-length ttRNH to specifically monitor the formation of the I_core+1 _intermediate. We determine that the ttRNH I_core+1 _intermediate is likely the intermediate populated prior to the rate-limiting barrier to global folding, in contrast to *E*. *coli* RNase H for which I_core _is the folding intermediate. This result provides new insight into the nature of the rate-limiting barrier for the folding of RNase H.

## Introduction

A fundamental goal in biology is to understand how the amino acid sequence encodes a protein’s energy landscape, defined as all accessible conformations of a protein, their associated energies, and the dynamics of inter-conversion between them [1]. Comparing structurally-homologous proteins gives us insight into what features of the landscape are dictated by native state topology versus what can be modulated by sequence [2].

The folding pathway, the sequence of partially folded intermediate states transiently populated as a protein folds, is a key feature of the energy landscape. (Other features of the landscape include partially folded intermediates that are not present on the folding pathway but which can nonetheless be reached by fluctuations at equilibrium.) There are a number of examples of proteins with the same native topology having differences in their folding pathways, from large differences in rates [3,4], to the presence or absence of folding intermediates [5,6] or differences in structural details of folding intermediates [7,8]. But it is particularly interesting to look for such differences between proteins whose native states have functionally different energy landscapes. For example, mesophilic and thermophilic protein homologs have very different stabilities with respect to temperature, a property very important for their function. How do differences in their energy landscapes relate to thermal stability? Interesting models for such a comparison are the RNase H homologs from the mesophile *E*. *coli* and from the thermophilic bacteria *Thermus thermophilus*.

**Fig 1:**
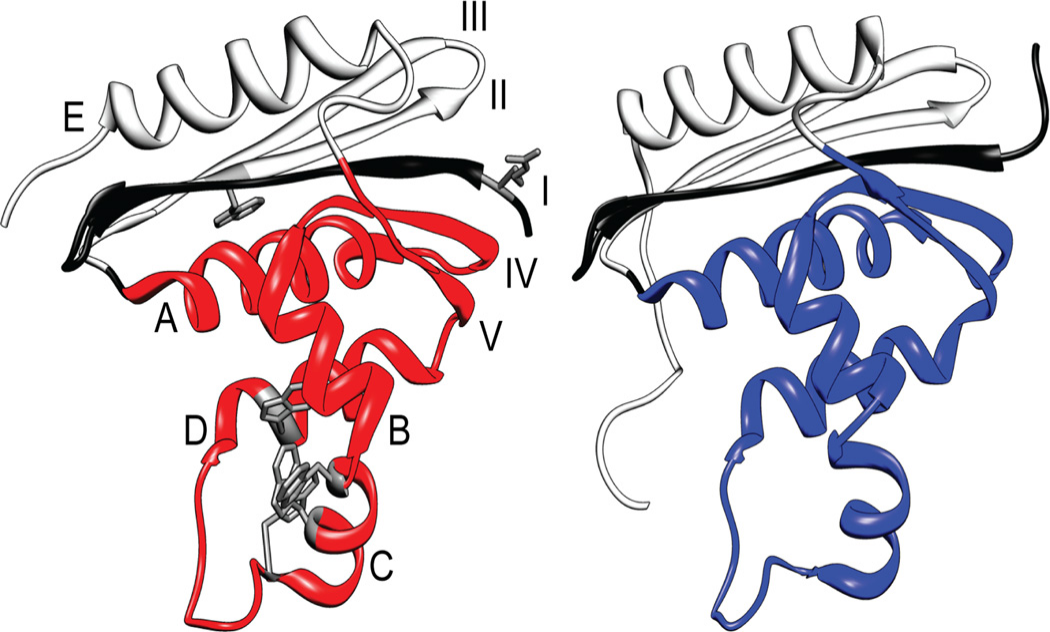
RNase H structures. *T*. *thermophilus* homolog on the left (pdb 1RIL) and *E*. *coli* homolog on the right (pdb 2RN2). The helices and strands are labeled on ttRNH (helices in letters, strands in Roman numerals). The structured region of the I_core_ intermediate model is depicted in red or blue. The additional residues included in the I_core+1_ intermediate model are highlighted in black. On ttRNH, all five tryptophan residues are highlighted in gray stick, as well as residue R4 on strand I.

*E*. *coli* RNase H (ecRNH) and *T*. *thermophilus* RNase H (ttRNH) have virtually identical native state topology [9,10] (Fig. 1), but different thermodynamic properties: ttRNH is more stable than ecRNH across a wide range of temperatures, and is active even at temperatures under which ecRNH is predominantly unfolded [11]. To investigate the source of ttRNH thermostability, both proteins have been studied using native-state (equilibrium) hydrogen exchange, and also kinetic experiments monitored by spectroscopy and pulse-labeling hydrogen exchange (all at 25°C) [12–15]. (In both cases, the “wild-type” protein is a cysteine-free version of true wild type [11,16,17].) The results suggested that both proteins have a similar distribution of stability across their structures, and that both populate a similar partially folded intermediate before the rate-limiting barrier to folding (within the 15 msec deadtime of the instrument). The current model of this intermediate is that it contains secondary structure in the contiguous region of the protein from helix A to strand V; this model is referred to as I_core_ (Fig. 1).

One big difference observed between the two homologs is that ttRNH has a lower ΔC_p_ (change in heat capacity between the unfolded and folded state). This serves to broaden the stability curve (ΔG_unf_ as a function of temperature), increasing the stability of ttRNH at all temperatures. It was inferred that the lower ΔC_p_ was due to residual structure in the unfolded state of ttRNH, which was confirmed using protein engineering studies and observed directly using calorimetry [18,19]. This result alone, however, cannot explain all the changes in the global unfolding energetics of the two proteins and does not address any other possible differences between their energy landscapes.

One possible difference between the ecRNH and ttRNH energy landscapes was suggested by a 2008 paper from the lab of Yawen Bai at the National Cancer Institute [20]. They propose that the structured region of the ttRNH folding intermediate includes strand I, which would make it very different from the ecRNH folding intermediate. (In the present work, we will refer to the intermediate populated immediately prior to the rate-limiting barrier to folding as “the” folding intermediate, though we know there are other intermediates on the folding pathway[21,22].) Bai and coworkers noted that previous ttRNH hydrogen exchange studies from our laboratory hint that strand I is possibly structured in kinetic and equilibrium intermediates [13,15]. They proposed a model where strand I is structured in the ttRNH folding intermediate in addition to the helical core, and they constructed a fragment mimic of the structured region of this model (to include strand I in the fragment they made a non-native junction where strand I is directly linked to the N-terminus of helix A, see Fig. 1). We will call this intermediate model I_core+1_. Their I_core+1_ fragment is well folded and they solved its NMR structure, showing that it looks like a subset of the native state structure.

The fact that strand I together with the region from helix A to strand V is well folded on its own indicates that I_core+1_ is a stable partially folded state of ttRNH. But we ask: is I_core+1_ truly the folding intermediate or is it populated elsewhere on the energy landscape (an equilibrium intermediate)? Is I_core_ also a stable partially folded state? And does ecRNH populate an I_core+1_ intermediate anywhere on its energy landscape? Answering these questions will address whether these features of the energy landscape of RNase H are defined by the native-state topology or whether the presence of these intermediates can be modulated by protein sequence (the two proteins contain ~50% sequence identity). In this work, we investigate these questions by making and characterizing fragment mimics of the putative I_core_ and I_core+1_ intermediates for both homologs (Fig. 1). The construction of the mimics follows a general approach where hydrogen exchange results are used to direct protein engineering [23], and where predicted unfolded regions are deleted [20,24,25].

By characterizing the protein fragments, we determine that ttRNH populates both I_core_ and I_core+1_ intermediates on its energy landscape, whereas ecRNH populates only the I_core_ intermediate. Additionally, we perform fluorescence resonance energy transfer (FRET) experiments to directly observe strand I docking onto the alpha-helical core to determine that ttRNH I_core+1_ is likely formed prior to the rate-limiting barrier to folding. This difference between the energy landscapes of ecRNH and ttRNH is the most dramatic observed between these two homologs to date, and changes our understanding of the major folding barrier for the RNase H model system. Future work will investigate whether the I_core+1_ intermediate plays a role in the thermal stability of the *T*. *thermophilius* RNase H homolog.

## Results

### Truncation mutants of *T*. *thermophilus* RNase H reveal multiple partially folded states on the energy landscape

A previous study from Yawen Bai’s lab demonstrated that ttRNH populates the I_core+1_ intermediate [20]. To determine whether ttRNH also populates the I_core_ intermediate, we created a fragment consisting of residues 43 to 122 (in this work, numbering for the ttRNH sequence is based on an alignment with the ecRNH sequence [11]). For comparison, we also re-created the Bai lab’s I_core+1_ fragment (residues -3 to 20 plus 42 to 122) but without the C-terminal hexahistine tag that was used in the original study.

**Fig 2:**
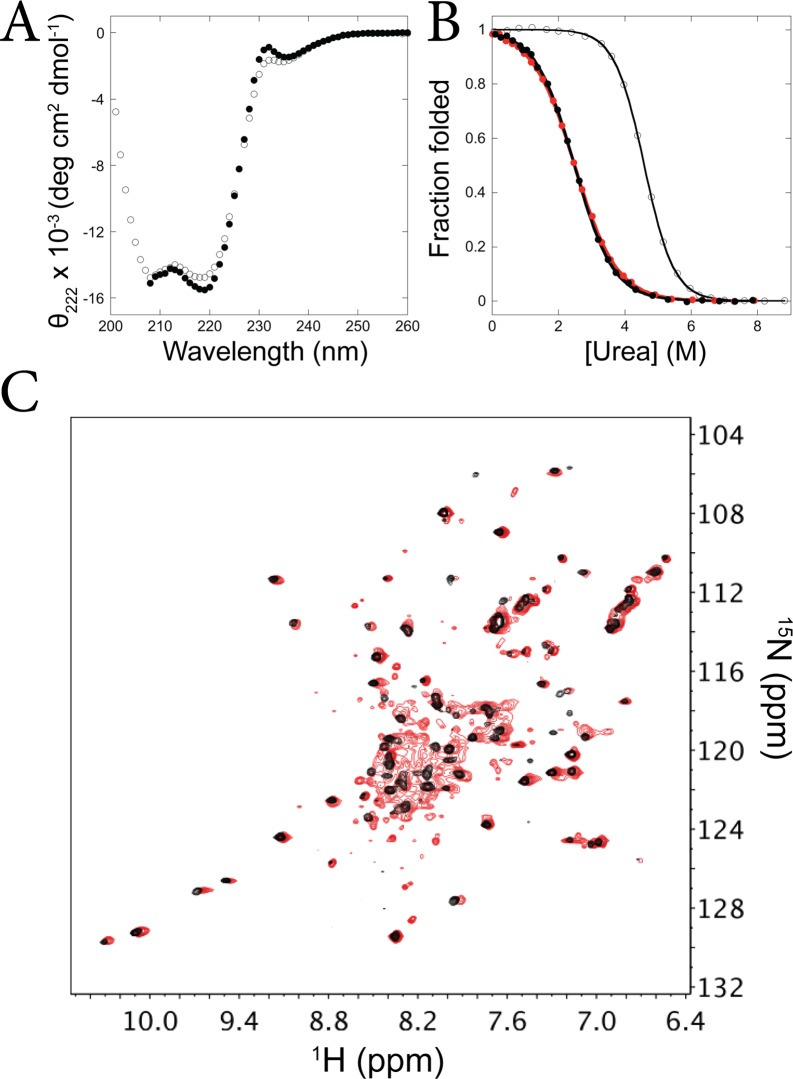
*T*. *thermophilus* RNase H populates two partially-folded states. (A) CD spectra of the ttRNH I_core_ fragment (black) measured at ~3.5 μM protein concentration and I_core+1_ fragment (white) measured at ~31 μM. (B) Representative equilibrium denaturation curves of the ttRNH I_core_ fragment at 3.6 μM (black) and 36 μM (red), and the I_core+1_ fragment (white), monitored by CD and normalized to fraction folded. (C) Overlay of ^1^H-^15^N HSQC spectra of the ttRNH I_core_ fragment measured at 54 μM (black) and 430 μM (red). The red peaks were shifted slightly to the right for easier comparison.

Circular dichroism (CD) studies indicate that both fragments are folded. The CD spectra show two minima near 208 nm and 222 nm, consistent with helix formation, and equilibrium urea-induced denaturation monitored by the CD signal at 222 nm show cooperative folding transitions (Fig. 2A-B, S1 Table). The denaturation curves can be fit using a two-state assumption and linear extrapolation model [26], yielding a ΔG_unf_ of 2.6 +/- 0.8 kcal/mol and an m-value of 1.00 +/- 0.09 kcal/mol/M for the I_core_ fragment and a ΔG_unf_ of 6.1 +/- 0.3 kcal/mol and an m-value of 1.34 +/- 0.08 kcal/mol/M for the I_core+1_ fragment. That the I_core_ fragment folds on its own indicates that the I_core_ partially folded intermediate is present on the ttRNH energy landscape in addition to the I_core+1_ intermediate.

### The ttRNH I_core_ fragment folds as a monomer with a K_d_ for dimerization of ~150 μM

**Fig 3:**
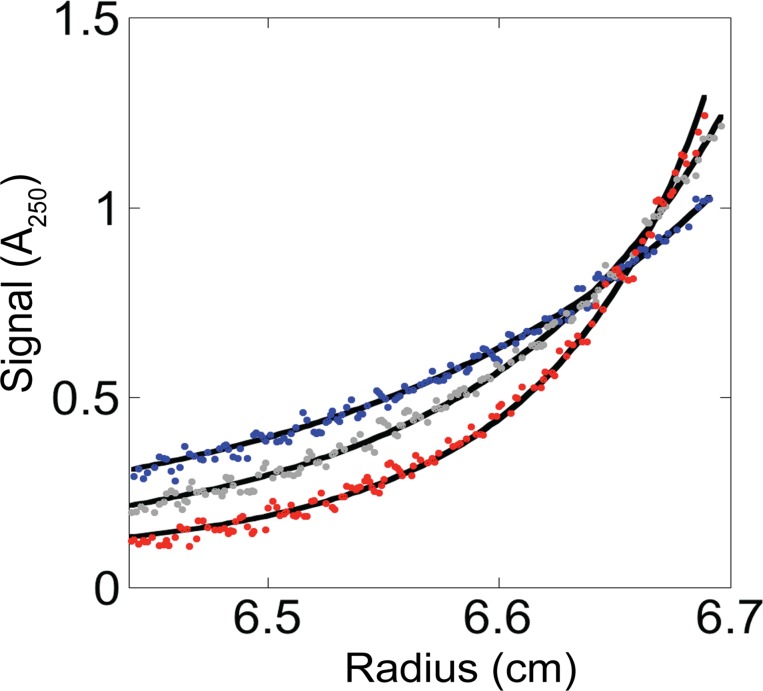
Equilibrium analytical ultracentrifugation of the *T*. *thermophilus* RNase H I_core_ fragment. Absorbance at 250 nm versus radius for a 50 μM sample at rotor speeds of 24,500 rpm (blue), 30,000 rpm (gray), and 37,000 rpm (red) was globally fit to a monomer-dimer equilibrium together with equivalent data from 25 μM and 100 μM samples.

To assure that the ttRNH I_core_ fragment is a monomer under our experimental conditions, we carried out equilibrium analytical ultracentrifugation (AUC). (It was previously determined that the ttRNH I_core+1_ fragment does not dimerize [20].) The AUC data was fit well by a monomer-dimer model, with a dissociation constant of ~150 μM (Fig. 3, S2 Table). Based on this K_d_, the CD samples (at 3–4 μM) are estimated to contain ~97% monomer. Therefore, we are effectively measuring properties of the monomer.

Additionally, we believe that the stability reported from the urea denaturation should accurately reflect monomer stability, since dimerization will be weakened by urea. To support this, the melt was repeated with a 10-fold higher protein concentration, at 36 μM, where ~85% monomer is expected in the 0 M urea sample (Fig. 2B, S1 Table). This melt was fit as before, and yielded a ΔG_unf_ of 2.3 kcal/mol and an m-value of 0.9 kcal/mol/M, consistent with results from the lower-concentration experiment.

### NMR suggests the interior of the ttRNH I_core_ fragment is closely packed

The I_core+1_ fragment was shown to be a well-folded structure using NMR, and in fact its structure was solved [20]. We wanted to determine whether the I_core_ fragment also has closely packed side chains, especially since it is less stable than the larger I_core+1_ fragment. We measured the ^1^H-^15^N heteronuclear single quantum coherence (HSQC) NMR spectrum of the I_core_ fragment at two protein concentrations—430 μM protein (~50% monomer expected) and 54 μM (~80% monomer expected)—in order to distinguish the signature of the monomer from the dimer. At high protein concentration, the spectrum shows peak dispersion, but also a cluster of significantly broadened peaks in the center. When the protein concentration is decreased, the broad, poorly-dispersed peaks disappear and the spectrum is dominated by sharp, well-dispersed peaks (Fig. 2C). This suggests that the monomer is well folded but that dimerization kinetics are on the right timescale to cause exchange broadening in peaks associated with the dimer.

### EcRNH does not populate the I_core+1_ intermediate

Having established that ttRNH populates two similar, well-folded intermediates, we asked if this was also true for the *E*. *coli* homolog. We previously constructed and characterized the ecRNH I_core_ fragment [25]; here, we constructed the I_core+1_ fragment (residues 1–20, 42–122).

**Fig 4:**
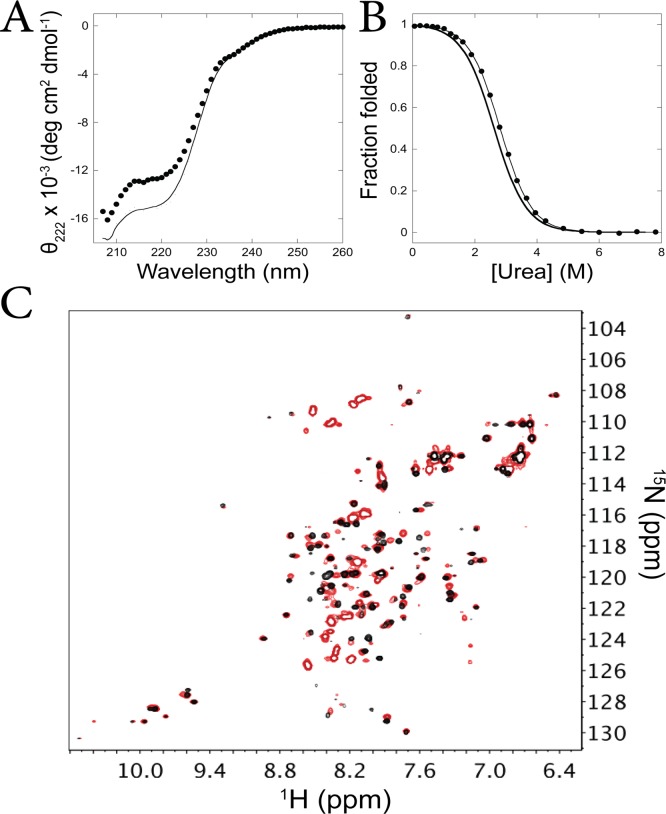
*E*. *coli* RNase H only populates the I_core_ intermediate. (A) CD spectrum of the ecRNH I_core+1_ fragment (black circles) compared to the ecRNH I_core_ fragment (line—from ref. [25]) measured at ~3.5 μM protein concentration. (B) Equilibrium denaturation of the ecRNH I_core+1_ fragment (black circles) compared to the ecRNH I_core_ fragment (line only—from ref. [25]), measured at ~3.5 μM protein concentration, normalized to fraction folded. (C) Overlay of HSQC spectra of the ecRNH I_core+1_ fragment (red) and the ecRNH I_core_ fragment (black—from ref. [25]) measured at ~100 μM.

Analysis by circular dichroism illustrates that the I_core+1_ fragment folds to a helical structure, with minima near 208 nm and 222 nm (Fig. 4A, S3 Table). Equilibrium denaturation with urea shows a cooperative transition, which can be fit using the same method as above, yielding ΔG_unf_ of 3.3 +/- 0.5 kcal/mol and an m-value of 1.2 +/- 0.1 kcal/mol/M (Fig. 4B, S3 Table). Both stability and m-value are within error of the equilibrium denaturation result for the ecRNH I_core_ fragment [25]. This result is in sharp contrast to the large stability difference (and m-value difference) between ttRNH I_core_ and I_core+1_ fragments, suggesting that the strand I residues are not contributing to the energetics and therefore likely not structured in the ecRNH I_core+1_ fragment. Hence, the presence of strand I does not affect the properties of the fragment and we conclude that ecRNH does not populate an I_core+1_ intermediate. This is further supported by previous work where full-length mimics of ecRNH I_core_ were made using mutations to selectively destabilize the native state [25]: the presence or absence of a mutation in strand I did not affect the stability of the full-length mimics.

Additional evidence is provided by the NMR HSQC spectrum of the ecRNH I_core+1_ fragment. Comparison to the spectrum of the ecRNH I_core_ fragment (measured previously [25]) shows that the majority of peaks are identical between the two spectra (Fig. 4C). (The matching peaks encompass both the I_core_ fragment peaks corresponding to monomer as well as those corresponding to a dimerized state, indicating that the ecRNH I_core+1_ fragment has a similar monomer-dimer equilibrium as the I_core_ fragment [25].) The major difference is found in the I_core+1_ fragment spectrum: a set of high intensity peaks with minimal dispersion along the ^1^H axis (centered at a ppm of ~8.3). This is the exact signature expected if strand I is unstructured in the I_core+1_ fragment.

### Strand I docks on the alpha helical core faster than global folding in ttRNH

*T*. *thermophilus* RNase H populates an I_core+1_ intermediate whereas *E*. *coli* RNase H does not, but where is this unique intermediate populated relative to the rate-limiting barrier to folding? To investigate this, we used FRET to directly monitor strand I contacting the alpha helical core during folding of the full-length protein. If I_core+1_ is populated prior to the rate-limiting barrier, we should observe that the strand I/helical core interaction occurs faster than global folding to the native state as monitored by CD.

Our FRET experiment was performed using intrinsic tryptophan fluorescence as the fluorescence donor and a thionitrobenzoate (TNB) label in strand I to quench fluorescence [27,28]. Wild-type ttRNH has five tryptophans: four in one group in the alpha helical core and one in strand II (Fig. 1). We made a conservative substitution (W22Y) in order to remove the tryptophan in strand II so that all the tryptophan fluorescence is present in the alpha helical core. Additionally a cysteine was engineered at position 4 (in place of a wild-type arginine) in order to attach the TNB label on the N-terminal end of strand I.

**Fig 5:**
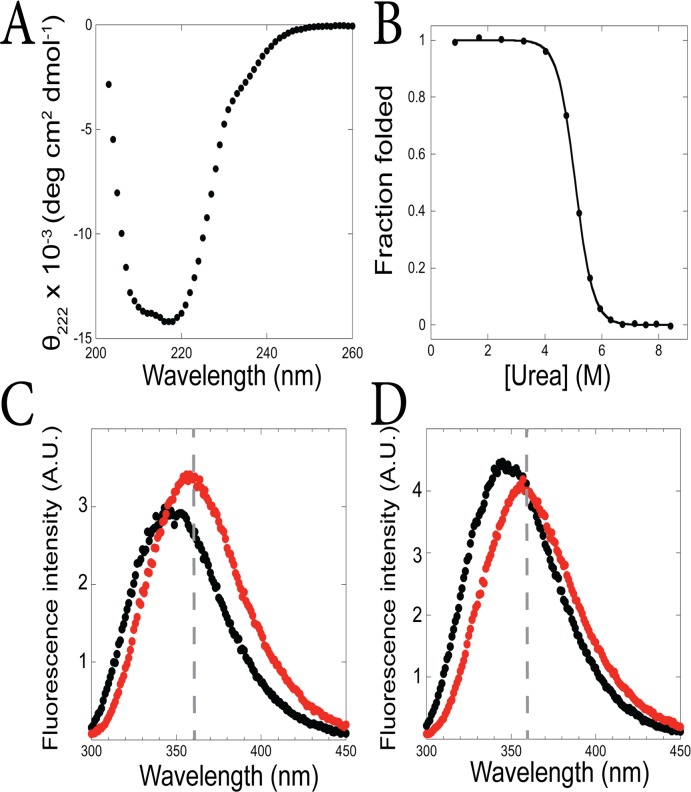
TNB-R4C/W22Y ttRNH is a good construct for monitoring strand I folding. (A) CD spectrum of TNB-labeled R4C/W22Y ttRNH exhibits the signature of well-folded RNase H. (B) Equilibrium denaturation of TNB-labeled R4C/W22Y ttRNH monitored by CD, fit to a two-state model and normalized to fraction folded. (C—D) Fluorescence emission spectra (with 295 nm excitation) of TNB-labeled (C) and unlabeled (D) R4C/W22Y ttRNH at 0 M urea (black) and 7 M urea (red). A gray dashed line marks emission at 360 nm.

The TNB-labeled R4C/W22Y construct was evaluated using CD to determine that the native structure and stability had not been too perturbed by the mutations and the TNB. The CD spectrum has a very similar shape as wild type, and equilibrium urea-denaturation yielded a ΔG_unf_ of 9.6 kcal/mol compared to the wild-type ΔG_unf_ of 12.8 kcal/mol [15], indicating that the protein likely has the same overall native structure with a decrease in stability (Fig. 5A-B, S4 Table). Additionally, we confirmed that the TNB label quenches tryptophan fluorescence in the native structure by measuring fluorescence emission spectra (with excitation at 295 nm) in both folded and unfolded conditions (Fig. 5C, S4 Table). The difference in signal is strongest near 360 nm, at which wavelength the unlabeled R4C/W22Y ttRNH protein shows almost no difference in fluorescence between folded and unfolded (Fig. 5D, S4 Table). Therefore, this is an ideal wavelength at which to monitor folding kinetics by FRET.

**Fig 6:**
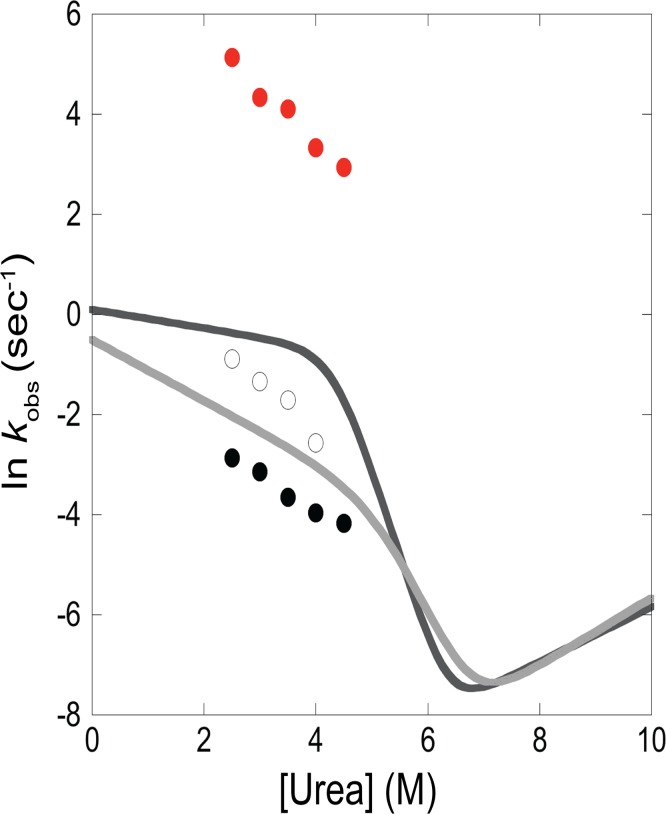
Strand I docking occurs much faster than global folding for TNB-R4C/W22Y ttRNH. Folding kinetics for the TNB-labeled protein were monitored by fluorescence emission at 360 nm (red dots) as well as by CD signal (black and hollow dots) over a range of final urea concentrations. For comparison, results of previously published WT ttRNH folding and unfolding kinetics are shown as lines [15]. There are two lines because a two-exponential decay is required to adequately fit WT folding. Likewise, for most of the CD folding experiments for TNB-R4C/W22Y ttRNH, a two-exponential decay is required to adequately fit the data (where adequate is determined by symmetrically-distributed residuals), hence two points are shown for these urea concentrations.

Re-folding of TNB-R4C/W22Y was monitored by both stopped-flow CD at 222 nm (a global probe of structure) and stopped-flow fluorescence at 360 nm (specifically monitoring formation of a strand I contact). Observed kinetics are very different using the two probes (Fig. 6, S5 and S6 Tables). Global folding measured by CD occurs on a timescale of seconds to minutes, very similar to that observed for the wild-type protein [15]. In contrast, when monitoring folding via FRET, kinetics are complete on a timescale of tens of milliseconds. This indicates that strand I attains native-like structure relative to the helical core on a timescale much faster than global folding. Thus, I_core+1_ is likely the intermediate formed prior to the rate-limiting barrier for *T*. *thermophilus* RNase H, as had been proposed previously [20].

## Discussion

In this work, we used fragment mimics of partially folded intermediate models to determine that *T*. *thermophilus* RNase H (ttRNH) populates the I_core_ and I_core+1_ intermediates, but *E*. *coli* RNase H (ecRNH) only populates an I_core_ intermediate. It had been previously determined that ttRNH can populate the I_core+1_ intermediate, i.e. that this subset of protein sequence can fold autonomously [20]. Here, we demonstrate that the ttRNH I_core_ fragment also folds, though with a significantly lower stability than the I_core+1_ fragment. In contrast, the ecRNH I_core_ and I_core+1_ fragments exhibit nearly the same stability, and the NMR HSQC spectrum of the ecRNH I_core+1_ fragment indicates that strand I is unstructured. Therefore, ecRNH strand I cannot dock onto the folded alpha helical core without further interactions with the rest of the protein. This is one of the clearest differences observed to date between the energy landscapes of these homologous proteins.

We then determined where on the ttRNH energy landscape is the I_core+1_ intermediate populated relative to the rate-limiting barrier to folding. Using a FRET experiment, we determined that strand I is likely structured in the ttRNH folding intermediate (i.e. preceding the rate-limiting barrier to folding) and therefore I_core+1_ is likely the ttRNH folding intermediate. This is a dramatic difference compared to the folding of the *E*. *coli* homolog.

The crystal structures of wild-type ecRNH and ttRNH yield few clues as to why the two proteins would have different behavior with respect to strand I (pdb 2RN2 *versus* 1RIL). In both native structures, strand I makes contact with core elements helix A and strand IV, in both cases with approximately the same number of interactions. The interface between strand I and strand IV in ttRNH shows a salt bridge (Arg4-Asp66) and an aromatic stacking interaction (Phe8-Tyr68) that are not present in ecRNH, but that is the extent of any obvious differences. The similarity of the two protein crystal structures highlights the need for experiments to capture the details of protein energy landscapes.

The present work provides insight into the nature of the rate-limiting barrier for RNase H folding. There are multiple hypotheses as to what is the most difficult step in RNase H folding. Historically, it had been thought that the barrier might be the packing down or tightening of molten tertiary interactions present in the folding intermediate. However, from recent work [25] as well as current results it appears that the structured region in the RNase H intermediate is likely well folded, invalidating this hypothesis. Another possibility is that the barrier is assembly of the beta sheet onto the well-folded alpha helical core (overcoming unfavorable conformational entropy). In this model, contact between strand I and the helical core would accompany global folding. Our FRET experiment illustrates this is not true for ttRNH and therefore is likely not the rate-limiting step.

We propose several possible scenarios for the slow step in global folding (though these scenarios are not mutually exclusive). One possibility is simply that the tertiary interactions needed to join the rest of the beta strands (I-III in the case of ecRNH and II and III in the case of ttRNH) to the natively-folded core form slowly, perhaps because of difficulty in attaining the correct geometry. This would correspond to slow docking of the final “foldon” onto the previously assembled native structural elements, as identified in previous work [21]. Another possibility is that non-native structure in the helical core of the intermediate results in an unfavorable interface for assembling the rest of the beta strands. The barrier could be a slow structural rearrangement in the core to form the native interface. (There is direct evidence for non-native structure in the helical core of the RNase H folding intermediate, though its exact nature is unknown [22].) Another possibility is that the “unfolded” region of the intermediate is itself trapped in a misfold. In this scenario, the slow step is a rearrangement within the residues that will form the beta strands in the native state. However, whatever the misfold, it can not result in the protection of hydrogen bonds, as there is no evidence for protection in this region of the protein until the rate-limiting step [14,21]. Future work will be needed to conclusively determine the nature of the slow, rate-limiting barrier.

The present work informs our understanding of RNase H folding, and sheds light on the interplay between topology and sequence in defining the folding pathway and the entire energy landscape. Future work evaluating the presence of the I_core+1_ intermediate along the evolutionary lineage between the *E*. *coli* and *T*. *thermophilus* homologs [29] could illuminate whether this is related to a general strategy for thermal stability.

## Materials and Methods

### Construction of RNase H variants

The ttRNH I_core_ and I_core+1_ fragments and the ecRNH I_core+1_ fragment were subcloned from pJH109 and pSM101, respectively. For the cloning of the I_core+1_ fragments, the N-terminal region of the sequence containing strand I was encoded on a primer. These constructs were all cloned into a pET27 vector, except for the ttRNH I_core+1_ fragment which was cloned into a modified pET28 vector with a TEV-cleavable hexahistidine tag. After TEV cleavage, a non-native glycine-histidine is left at the N-terminus of the ttRNH I_core+1_ fragment.

The full-length ttRNH variant was created in the pSV272 vector, with a TEV-cleavable hexahistidine-tagged MBP fusion at the N-terminus of the RNase H gene. Mutations were created using Quikchange.

### Protein expression and purification

Expression and purification of the fragments is as previously described for the ecRNH I_core_ fragment [25], with the following exception. The ttRNH I_core+1_ fragment was purified from the soluble fraction, using a Ni column and then TEV cleavage prior to purification with a Capto S column.

Expression of ^15^N labeled protein was done by initial growth in LB with a switch to M9 media with ^15^NH_4_Cl as the sole nitrogen source prior to induction for three hours by IPTG. The labeling efficiency was ~90% as evaluated by mass spectrometry.

Expression of the MBP-ttRNH fusion protein was performed as previously [17] except using Rosetta2(DE3)pLysS cells and kanamycin. For purification, cell pellets were lysed by sonication, cell debris was removed by centrifugation and the soluble fraction was first purified using a Ni column. After overnight TEV-cleavage, fractions containing ttRNH are purified in a final step using a Heparin column (which removes the free MBP very efficiently).

### TNB labeling

Labeling of the single cysteine in the R4C/W22Y ttRNH variant was accomplished by incubating protein in 6 M GdmCl, 20 mM Tris, pH 8.3, and 250 μM EDTA with a 50x molar excess of DTNB at room temperature for 30 minutes (the protein had been prepared with a Zeba spin desalting column to remove reducing agent). Another Zeba column was used to exchange the labeled protein into unfolding buffer for kinetic experiments (7 M urea, 20 mM sodium acetate, pH 5.5, and 50 mM potassium chloride) and simultaneously remove free dye. Mass spectrometry indicated that the labeling efficiency was ~100%.

### Equilibrium experiments

All experiments were performed at room temperature, with the following buffer conditions: 20 mM sodium acetate, pH 5.5, and 50 mM potassium chloride. CD experiments were measured on an Aviv 410 CD spectropolarimeter in a cuvette with a 1-mm or 1-cm pathlength, as appropriate to the protein concentration. For each construct, at least one equilibrium denaturation was performed after incubating individual samples overnight. The rest were performed with shorter incubations, some using a titrator with a 5-min equilibration time between samples. (The exception is that all samples for the full-length ttRNH variant were incubated overnight.) The results were consistent at all incubation times. 95% confidence intervals are based on the average of 3–5 experiments. NMR experiments were recorded on a Bruker Avance II 900-MHz spectrometer, as described previously [25]. Equilibrium ultracentrifugation experiments were performed with a Beckman XL-I analytical ultracentrifuge, as described previously [25].

### Kinetic experiments

Kinetics monitored by CD at 222 nm were performed with either an Aviv 202 stopped-flow instrument using an 11-fold dilution of sample or an Aviv 410 with a 1-cm pathlength cuvette using a 30-fold dilution of sample. The dead time for the stopped-flow experiments is 18 milliseconds and for the manual mixing experiments is ~15 seconds. For experimental conditions where kinetics were monitored by both stopped flow and manual mixing (3.5 M, 4 M and 4.5 M urea) the two data sets were fit simultaneously to obtain the rate constants that best described the data. (For the combined data sets, final urea concentrations were within 0.1 M of each other.) Only the 4.5 M data was fit with a single exponential. All other data sets were fit with two-exponentials in order to achieve symmetrically distributed residuals.

Kinetics monitored by fluorescence were performed on a Biologic SFM-400 stopped-flow instrument. Kinetics were initiated using a 10-fold dilution into the FC-15 cuvette, with a 250 uL shot volume and 7 mL/sec total flow speed, resulting in a dead time of 5.2 milliseconds. All data were fit to single exponentials.

Data were fit using IgorPro.

## Supporting Information

S1 Table Data obtained from CD analysis of ttRNH fragments. (A) CD spectra of I_core_ and I_core+1_ (MRE *versus* wavelength). (B) Representative equilibrium melts measured by CD (Fraction folded *versus* urea concentration) for I_core_ (at two protein concentrations) and I_core+1_.
(XLSX)

S2 Table Analytical ultracentrifugation data for ttRNH I_core_. (XLSX)

S3 Table Data obtained from CD analysis of the ecRNH I_core+1_ fragment. (A) CD spectrum (MRE *versus* wavelength). (B) Representative equilibrium melt measured by CD (Fraction folded *versus* urea concentration).
(XLSX)

S4 Table Equilibrium data measured for TNB-R4C/W22Y ttRNH. (A) CD spectrum (MRE *versus* wavelength). (B) Equilibrium melt measured by CD (Fraction folded *versus* urea concentration). (C) Fluorescence emission spectra of TNB-R4C/W22Y ttRNH in folded and unfolded conditions. (D) Fluorescence emission spectra of R4C/W22Y ttRNH in folded and unfolded conditions.
(XLSX)

S5 Table Rate constants determined for TNB-R4C/W22Y folding as monitored by CD and fluorescence at multiple urea concentrations. (XLSX)

S6 Table Raw kinetic data for TNB-R4C/W22Y ttRNH folding. (A) Folding monitored by stopped-flow mixing and fluorescence. (B) Folding monitored by stopped-flow mixing and CD. (C) Folding monitored by manual mixing and CD.
(XLSX)
